# Advanced Electrochemical Monitoring of Carbendazim Fungicide in Foods Using Interfacial Superassembly of NRPC/NiMn Frameworks

**DOI:** 10.3390/bios14100474

**Published:** 2024-10-02

**Authors:** Shakila Parveen Asrafali, Thirukumaran Periyasamy, Seong Cheol Kim, Jaewoong Lee

**Affiliations:** 1 Department of Fiber System Engineering, Yeungnam University, Gyeongsan 38541, Republic of Korea; shakilaasraf@gmail.com (S.P.A.);; 2 School of Chemical Engineering, Yeungnam University, Gyeongsan 38541, Republic of Korea

**Keywords:** polybenzoxazine, porous carbon, carbendazim, sensor

## Abstract

A simple, sensitive and reliable sensing system based on nitrogen-rich porous carbon (NRPC) and transition metals, NRPC/Ni, NRPC/Mn and NRPC/NiMn was developed and successfully applied as electrode materials for the quantitative determination of carbendazim (CBZ). The synergistic effect of NRPC and bimetals with acceptable pore structure together with flower-like morphology resulted in producing a highly conductive and interconnected network in NRPC/NiMn@GCE, which significantly enhanced the detection performance of CBZ. The electrochemical behavior investigated by cyclic voltammetry (CV) showed improved CBZ detection for NRPC/NiMn, due to the controlled adsorption/diffusion process of CBZ by the NRPC/NiMn@GCE electrode. The influences of various factors such as pH, NRPC/NiMn concentration, CBZ concentration and scan rate were studied. Under optimal conditions, 0.1 M phosphate-buffered saline (PBS) with a pH of 7.0 containing 30 µg/mL NRPC/NiMn, a favourable linear range detection of CBZ from 5 to 50 µM was obtained. Moreover, a chronoamperometric analysis showed excellent repeatability, reproducibility and anti-interfering ability of the fabricated NRPC/NiMn@GCE sensor. Furthermore, the sensor showed satisfactory results for CBZ detection in real samples with acceptable recoveries of 96.40–104.98% and low RSD values of 0.25–3.45%.

## 1. Introduction

In the modern world, there is tremendous growth in the agricultural field by incorporating new methods to increase the yield, to keep it fresh for a long time and to produce hybrid varieties. Hence, to cope with these tasks, pesticides in various forms (herbicide, insecticide, fungicide) have been used in a considerable amount. Carbendazim (CBZ), chemically named methyl-2-benzimidazole carbamate, is a common pesticide that has been widely used in agriculture during pre- and post-harvesting to prevent damage to fruits and cereal crops (such as wheat, rice and cotton) affected by fungal diseases such as spot, mildew, mold, rot and scorch [1,2,3,4,5]. CBZ found in fruit peels and cereals is a potential human carcinogen, and if consumed by humans can lead to liver disease and chromosome aberration. CBZ contains a benzimidazole ring that cannot be degraded easily and its extensive use causes the residues to remain in food, water and soil, causing serious issues to human health and the environment. In general, a maximum residue level (MRL) of 0.01 mg/kg is applicable to all pesticides [6]. And therefore, designing a simple, fast and sensitive detection method to accurately measure the CBZ residues is in high demand [6,7,8,9].

There are several traditional analytical methods used for the detection of CBZ, including high-performance liquid chromatography (HPLC), gas chromatography–mass spectrometry (GC-MS), ultraviolet–visible spectroscopy (UV-Vis) and fluorescence spectroscopy. Nonetheless, all these techniques are time consuming. And so, electrochemical sensing has received much attention recently due to its high sensitivity, rapid analysis, low cost, user friendliness and portability [10,11,12,13,14,15]. Carbon-based materials such as graphene, carbon black and carbon nanotubes (CNTs) and metals including metal nanoparticles, metal oxides and transition metal oxides (TMOs) are widely used as electrochemical sensing materials for the detection of trace level of pesticides in food including fruits, vegetables and crops [16,17,18,19,20,21,22].

Gao et al. [23] used nano-porous gold (NPG) as an electrochemical sensor for the detection of two different pesticides, methyl parathion (MP) and carbendazim (CBM). The prepared NPG/GC (glassy carbon) electrode exhibits high sensitivity for MP (186.53 µA mM^−1^ cm^−2^) and CBM (484.51 µA mM^−1^ cm^−2^), and low detection limits (0.02 mM for MP and 0.24 mM for CBM). Additionally, it also provides high specificity, selectivity and anti-interference capabilities. Transition metal oxides and metal nanoparticles exhibit good electrical and photocatalytic properties, as they possess different shapes, structures, stability and large surface area. But their major drawback is their wide band gap, which reduces their conductivity and finally leads to pulverization of the electrode film. Hence, to overcome this issue, the metal particles are used along with a carbon matrix.

Ji et al. [21] prepared a novel electrochemical sensor based on multiwalled carbon nanotubes (MWNTs) and Au nanoparticles (AuNPs) on molecularly imprinted polymer (MIP) membranes for the clinical diagnosis of cholesterol. The MIP sensor showed a linear response range between 10^−13^ and 10^−19^ mol L^−1^ and the limit of detection (LOD) was found to be 3.3 × 10^−14^ mol L^−1^. Anshori et al. [18] synthesized a bio-sensing material based on functionalized multi-walled carbon nanotube/silver nanoparticles (f-MWCNT/AgNPs) for the detection of dopamine exhibiting an LOD value of 0.2778 mM in the linear range of 0–8 mM. Wang et al. [20] proposed a low-cost ultrasonic assisted strategy and prepared graphitized and carboxylated carbon nanotubes along with cerium oxide, GR-MWCNT-COOH@CeO_2_/GCE sensor for the detection of methyl parathion with a lower LOD value of 0.0285 mM. Wu et al. [22] prepared palladium-multiwalled carbon nanotube composites (Pd-MWCNTs) as a sensing platform for acetaminophen showing an acceptable linear response between 0.5 and 100 µM with a detection limit of 0.13 µM. Gao et al. [17] synthesized gold and zirconia nanocomposite-modified graphene nanosheets (Au-ZrO_2_-GNs/GCE) for the electrochemical detection of methyl parathion showing a very low LOD of 1 ng mL^−1^.

Based on the above-mentioned reports, we proposed the combination of carbon material and transition metal oxide for the detection of carbendazim. The carbon material, namely, nitrogen-rich porous carbon (NRPC), was synthesized from polybenzoxazine and the transition metal inclusion into NRPC, i.e., NRPC/Ni, NRPC/Mn and NRPC/NiMn, was carried out with subsequent methods involving carbonization, activation and hydrothermal reactions. We believe that the synergistic effect produced from the combination of NRPC and transition metal(s) could improve the electrochemical sensing performance for the detection of CBZ.

## 2. Materials and Methods

### 2.1. Materials

Phenylethylamine, hydroquinone and paraformaldehyde were procured from Sigma-Aldrich, Seoul, Republic of Korea, while melamine sponge, stearic acid, potassium hydroxide (KOH), sodium hydroxide (NaOH), dimethyl sulfoxide (DMSO), nickel nitrate hexahydrate, manganese nitrate tetrahydrate, polyvinylidene fluoride (PVDF) and N, N-dimethylformamide (DMF) were obtained from Duksan Chemicals Co., Ltd., Gyeonggi-do, Republic of Korea. These chemicals were used as received without any further purification.

### 2.2. Synthesis of the NRPC/NiMn Hierarchical Structure

The process for synthesizing both monometallic and bimetallic NRPC-supported materials is outlined as follows: First, separate 250 mL solutions of individual metal salts, specifically Mn(NO_3_)_2_·4H_2_O and Ni(NO_3_)_2_·6H_2_O, were prepared at a concentration of 6 mM. Additionally, a mixed metal salt solution was created by combining Mn(NO_3_)_2_·4H_2_O and Ni(NO_3_)_2_·6H_2_O at the same concentration. These three solutions referred to as A (Mn solution), B (Ni solution) and C (mixed Mn/Ni solution) were each taken in separate containers. To each of these solutions (A, B and C), 10 mg of NRPC was introduced and thoroughly dispersed. Following this, 18 mM of NH_4_F was added to the dispersed solutions, which were then subjected to ultrasonication for one hour to ensure thorough mixing and dispersion. In parallel, a basic solution was prepared by dissolving 12 mM of NaOH and 30 mM of Na_2_CO_3_ in water. This basic solution was then added dropwise to the previously ultrasonicated mixtures under continuous stirring. The stirring was maintained rigorously for four hours to facilitate the reaction and the formation of the desired products.

After stirring, the resultant suspensions were left to age at room temperature overnight. Following this ageing process, the suspensions were transferred to a sealed autoclave and heated to 90 °C for 12 h to promote further reaction and crystallization. The final products were obtained by washing the suspensions with water and drying them at room temperature, resulting in NRPC-supported transition metal oxides. The entire synthesis is summarized in Scheme S1.

## 3. Results and Discussions

**Figure 1:**
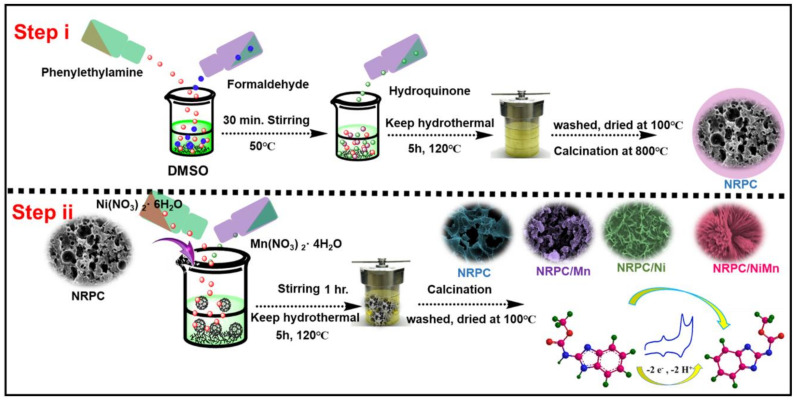
Synthesis of NRPC, NRPC/Mn, NRPC/Ni and NRPC/NiMn.

The structure and morphology of the synthesized materials (Figure 1), NRPC, NRPC/Mn, NRPC/Ni and NRPC/NiMn, were characterized and analyzed with different methods including Raman, XRD, BET, XPS, SEM and TEM analyses. The Raman spectra of the prepared materials are given in Figure S1a. The NRPC material shows two bands at 1354 and 1586 cm^−1^, respectively, denoting the ‘D band’ and ‘G band’. The ‘D band’ is due to the degree of disorderedness and the ‘G band’ is due to the degree of graphitization. The other materials, NRPC/Mn, NRPC/Ni and NRPC/NiMn, show a third band at 534 cm^−1^, due to M-O-M bonds, in addition to the D and G bands. The ratio of the intensity of the ‘D band’ (I_D_) to the intensity of the ‘G band’ (I_G_) gives an idea of the extent of disorderedness produced. It could be observed that the I_D_/I_G_ value is larger for NRPC/NiMn, indicating increased disorderedness [23,24].

The XRD patterns of the prepared materials are given in Figure S1b. The pristine carbon material, NRPC, shows two diffraction peaks at 24.2 and 44.6°, respectively, corresponding to the (002) and (001) planes of the carbon materials. NRPC/Mn, NRPC/Ni and NRPC/NiMn show additional diffraction patterns corresponding to the (003), (101), (012), (015) and (018) planes of the rhombohedral phase. The property of the prepared materials was analyzed with BET analysis. Figure S1c,d display the N_2_ adsorption/desorption isotherm and pore size distribution (PSD) of the given materials. The N_2_ adsorption/desorption isotherms show type IV isotherm with an H_3_ hysteresis loop denoting mesoporous materials. The PSD curves indicate the presence of both micro- and mesoporous materials with pore diameter ranging between 2 and 50 nm [25,26].

**Figure 2:**
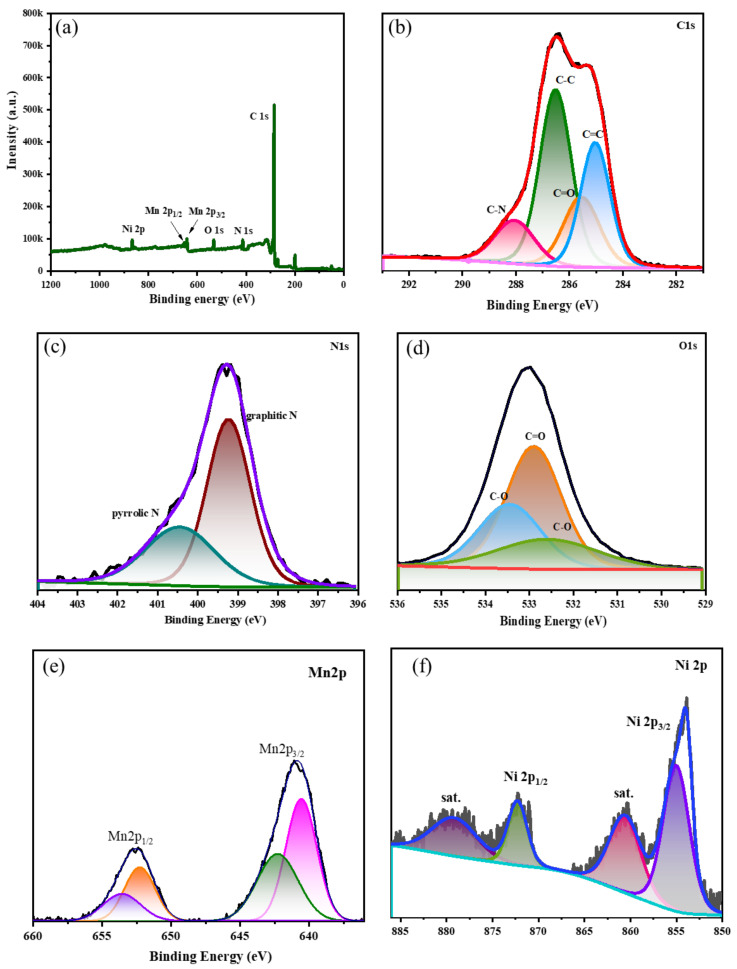
XPS spectrum of NRPC/NiMn showing the (**a**) survey spectrum and (**b**–**f**) deconvoluted spectrum for each element.

X-ray photoelectron spectroscopy (XPS), a powerful analytical technique, was employed to unravel the elemental makeup and intricate chemical interactions occurring on the surface of NRPC/NiMn materials. This investigation yielded invaluable data, shedding light on the material’s fundamental characteristics and paving way for future advancements. Figure 2a–f showcase high-resolution XPS spectra, akin to chemical fingerprints, for C 1s, N 1s, O 1s, Mn *2p* and Ni *2p*. These spectra meticulously detail the symphony of elements present within NRPC/NiMn, offering a comprehensive perspective on its building blocks. Notably, the absence of any extraneous peaks underscores the exceptional purity of both NRPC and NRPC composite materials. To delve deeper into the interplay between various atoms, the XPS survey scan was deconvoluted and is shown in Figure 2b–f. This scan unveils distinct peaks corresponding to each element—carbon (C), nitrogen (N), oxygen (O), manganese (Mn) and nickel (Ni)—at characteristic binding energies of approximately 285, 399, 533, 643 and 860 eV, respectively. These unique energy signatures act as identifiers for each element, akin to a chemist’s fingerprint library. Zooming in on the C 1s spectrum (Figure 2b) reveals a captivating story. Four distinct peaks emerge, each representing a specific chemical environment surrounding the carbon atoms. These peaks meticulously map various carbon-based bonds, including hydrocarbon chains (C–C and C=C) at 286.5 and 285.2 eV, carbon–nitrogen bonds (C–N) at 288.2 eV and carbons linked to oxygen-containing groups (C=O) at 285.6 eV. By deciphering these peaks, scientists can unveil the intricate molecular structure and composition of the material. Similarly, the XPS technique sheds light on the diverse configurations of nitrogen atoms within NRPC/NiMn. The N 1s spectrum unveils a fascinating array of peaks at varying binding energies, each corresponding to a specific type of nitrogen species. Graphitic and pyrrolic N can be identified by their unique energy signatures at 399.4 and 400.5 eV, signifying the presence of a rich tapestry of nitrogen functionalities within the material. Interestingly, the nitrogen species originating from the polybenzoxazine precursor are known to exhibit enhanced electrochemical activity, contributing to a significant boost in capacitance, particularly in acidic environments. XPS analysis also unveils the intriguing interplay between oxygen and carbon atoms. The O 1s spectrum resolves the oxygen bonding into distinct peaks at 532.9, 533.5 and 532.5 eV, each representing quinone and phenolic hydroxyl groups. These functional groups play a critical role in defining the material’s properties. Notably, phenolic hydroxyl groups are particularly noteworthy for their ability to facilitate high pseudo-capacitance through reduction reactions, ultimately enhancing the material’s overall performance [27,28]. Delving deeper, the Mn *2p* spectrum unveils a captivating story of manganese. The Mn *2p* spectrum (Figure 2e) reveals four peaks showing different states of Mn. The peaks at 641.5 and 652.5 eV correspond to Mn^2+^ *2p_3/2_* and Mn^2+^ *2p_1/2_*, and the peaks at 642.5 and 653.5 eV correspond to Mn^3+^ *2p_3/2_* and Mn^3+^ *2p_1/2_*. This analysis is crucial for understanding the electrochemical behavior of manganese in the material. Similarly, the Ni *2p* spectrum (Figure 2f) shows peaks corresponding to Ni^2+^ *2p_3/2_* and Ni^2+^ *2p_1/2_* at 855.0 and 872.5 eV, respectively, along with satellite peaks at 861.5 and 880.0 eV, signifying different nickel species [29]. These peaks provide insight into the oxidation states of nickel within the material, which are important for its catalytic and electrochemical properties.

**Figure 3:**
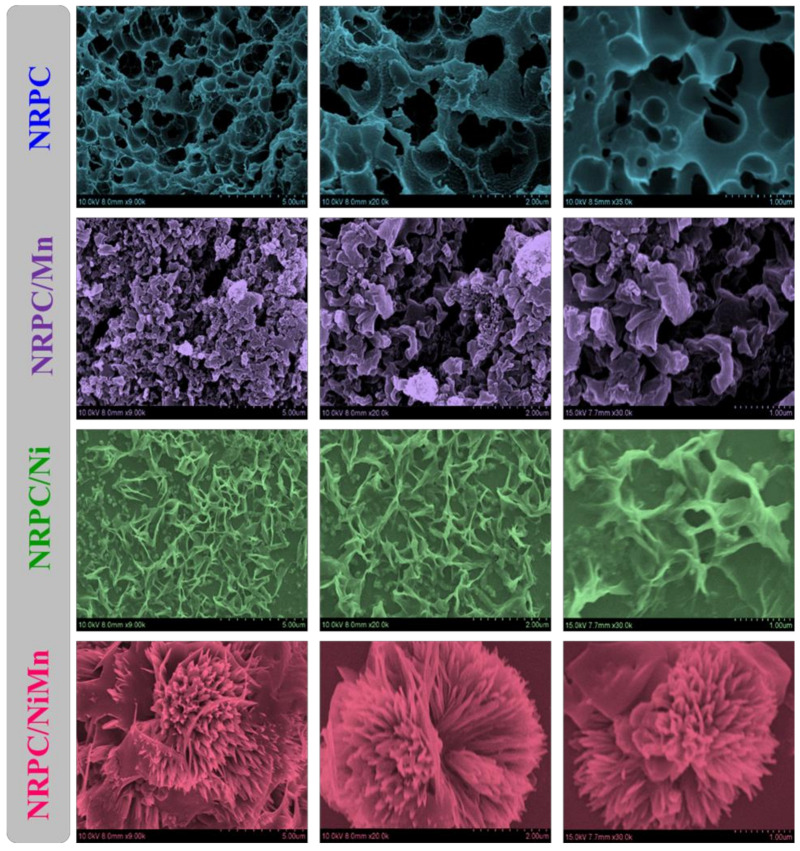
SEM images of NRPC, NRPC/Mn, NRPC/Ni and NRPC/NiMn at different magnifications.

Scanning electron microscopy (SEM) was employed to gain a deeper understanding of how the microscopic structures of different materials evolve. These materials included porous carbon (NRPC), and composites where NRPC was combined with manganese (Mn), nickel (Ni) or both Mn and Ni (NRPC/NiMn). The resulting magnified images are presented in Figure 3. At its core, NRPC resembled a maze (labyrinthine network) riddled with numerous holes (pores) and empty spaces (voids). This intricate framework included few large pores (macropores) of different sizes scattered amongst the voids. Interestingly, the overall structure appeared somewhat compressed, hinting at a potential collapse. When manganese (Mn) was incorporated into NRPC (NRPC/Mn), a remarkable transformation occurred. The initially observed maze-like structure gave way to unique flake-like shapes with wider ends. In contrast, adding nickel (Ni) to NRPC (NRPC/Ni) produced a distinct “crushed petal” morphology with narrow, pointed ends (spike-like). It is worth noting that these petal formations seemed to be intricately woven into the existing porous carbon matrix of NRPC. The combination of both Mn and Ni (NRPC/NiMn) yielded particularly fascinating results.

The presence of these two elements triggered the formation of numerous interconnected petal-like structures. Remarkably, these petals exhibited a vertical growth pattern, eventually merging to create a hierarchical 3D flower-like architecture. This growth pattern implied a gradual building process, where an increasing number of petals densely packed together to form a cohesive structure. At the centre of this 3D flower-like structure resided a core containing empty spaces (voids). This core acted as a central hub for the petals, providing crucial support and maintaining the structural integrity of the entire 3D framework. This inner core played a critical role in ensuring the stability of the flower-shaped structure. Additionally, the interconnected voids within the core offered a multitude of electroactive sites, which are essential for storing ions in supercapacitor electrodes [23,24].

**Figure 4:**
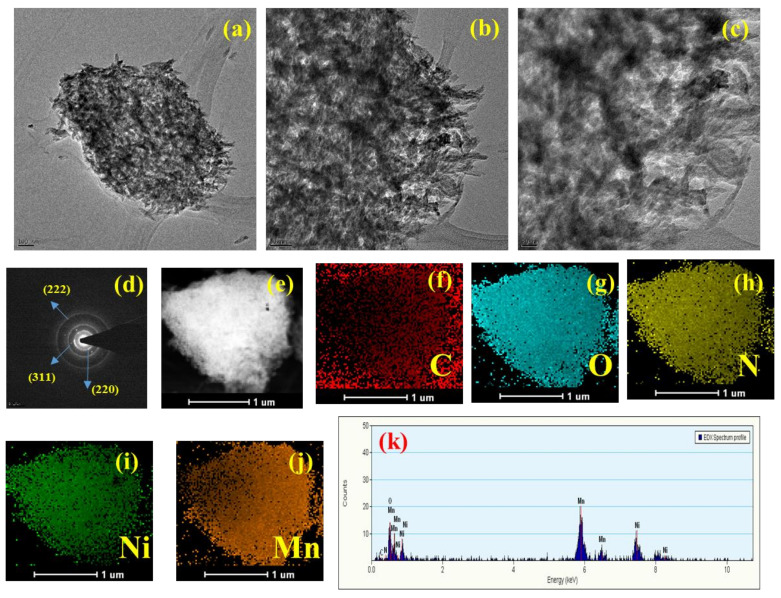
(**a**–**c**) TEM images of NRPC/NiMn, (**d**) SAED pattern, (**e**–**j**) elemental mapping and (**k**) EDX spectrum.

To investigate the morphology and elemental composition of the bimetallic species embedded within the porous carbon matrix (NRPC/NiMn), we employed Transmission Electron Microscopy (TEM) techniques. The TEM image offers a high-resolution view on a nanoscale (Figure 4a–c), clearly revealing the porous carbon structure hosting well-dispersed clusters of metallic nanoparticles. This visualization provides direct evidence for the successful integration of the desired bimetallic component within the carbon matrix. Further confirmation of the material’s composition came from SAED and Energy-Dispersive X-ray Spectroscopy (EDX) mapping (Figure 4d–k). The diffraction rings corresponding to different planes, (220), (311) and (222), are evident from the SAED pattern. This technique identified a broad spectrum of elements, including carbon (C), oxygen (O), nitrogen (N), nickel (Ni) and manganese (Mn). This comprehensive elemental analysis aligns perfectly with our initial hypothesis. The presence of oxygen and nitrogen signifies their successful incorporation into the porous carbon scaffold, likely facilitated by the use of Pbz as the carbon source. Moreover, the detection of both nickel and manganese elements confirms their intended integration into the carbon framework. This successful incorporation of all targeted elements from the Pbz precursor and bimetals represents a crucial step forward in tailoring the material’s property for enhanced electrochemical performance. This innovative approach, combining Pbz-derived carbon with embedded bimetallic species, establishes a valuable benchmark for future research efforts. It paves the way for further exploration aimed at optimizing the performance and efficiency of energy-storage devices like supercapacitors. This advancement has the potential to significantly impact various technological applications that rely on high-performance energy-storage solutions.

## 4. Electrochemical Characterization

### 4.1. Electrochemical Behavior of the Materials towards CBZ

**Figure 5:**
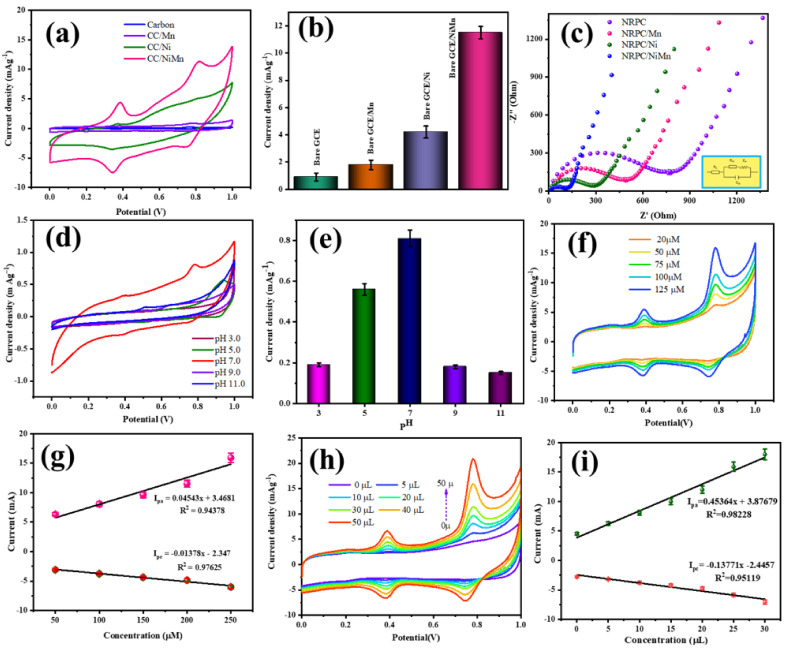
Electrochemical studies showing (**a**,**b**) CV, (**c**) EIS, (**d**,**e**) effect of pH, (**f**,**g**) effect of material concentration and (**h**,**i**) effect of analyte concentration of the prepared materials.

The electrochemical sensing behavior of the synthesized materials towards carbendazim was analyzed by CV measurement. The working electrode was prepared by coating 100 µM of the synthesized material onto the glassy carbon electrode and drying completely. The platinum electrode acts as the current collector and Ag/AgCl acts as the reference electrode. The CV measurements were found by immersing these electrodes in PBS solution (0.1 M, pH = 7.0) with the addition of 30 µM of the analyte (CBZ). Figure 5a shows the electrochemical behavior of NRPC, NRPC/Mn, NRPC/Ni and NRPC/NiMn towards CBZ. As could be seen, two oxidation peaks were observed, one at 0.4 V due to carbofuran and the other at 0.8 V due to carbendazim. The intensity of the oxidation peak for the detection of CBZ was found to be 1.0 mA g^−1^ for NRPC, 2.0 mA g^−1^ for NRPC/Mn, 4.2 mA g^−1^ for NRPC/Ni and 11.8 mA g^−1^ for NRPC/NiMn (Figure 5b). In contrast, NRPC and NRPC/Mn show weak oxidation peaks, whereas NRPC/Ni shows a moderate oxidation peak and NRPC/NiMn shows a well-defined oxidation peak. The detection of carbendazim happens in this way: carbendazim, being a highly polar molecule, interacts with NRPC through an electrostatic interaction (π-π interaction) and hydrogen bonding interactions. The enhanced signal obtained for NRPC/NiMn is due to the presence of numerable electroactive sites produced from both hetero-atom doping and bimetal inclusion in the carbon framework. Thus, the synergistic effect produced by NRPC and bimetallic species improved the morphology of NRPC/NiMn (Figure 3), showing a properly integrated flower-like morphology, in which the carbon framework forms a base structure, upon which the bimetallic species were grown in the form of petals, together resembling a fully bloomed flower [1,30,31]. Moreover, the bimetallic species are distributed uniformly in the carbon matrix without any agglomeration. All these factors result in increased conductivity of NRPC/NiMn and therefore show increased current density for CBZ detection.

### 4.2. Impedance Analysis

The interfacial characteristics including conductive, insulative and diffusive properties of the NRPC-based electrodes were examined by impedance analysis. Figure 5c represents the EIS spectra of NRPC, NRPC/Mn, NRPC/Ni and NRPC/NiMn electrodes in 0.1 M PBS solution containing 30 µM of carbendazim. The circuit diagram fits with the Randle’s equivalent circuit, which includes electrolyte resistance (R_s_), charge-transfer resistance (R_ct_) and Warburg impedance (Z_w_). All the prepared electrodes show a semicircle with a tail that extends further. The starting point of the semicircle represents the resistance of the electrolyte; the diameter of the semicircle gives the charge-transfer resistance; and the extended line from the semicircle indicates the Warburg impedance. Among these, the charge-transfer resistance is important, as it gives information about the conductive/insulative property at the electrode/electrolyte interface. As could be observed, the R_ct_ was found to be 760 Ω for NRPC, 490 Ω for NRPC/Mn, 300 Ω for NRPC/Ni and 120 Ω for NRPC/NiMn. In contrast, a very low R_ct_ value of 120 Ω was obtained for the NRPC/NiMn electrode, indicating that the hetero-atom doping (nitrogen and oxygen) and the inclusion of bimetallic transition elements creates an excellent electron-transfer medium and enhanced the conductivity of the NRPC materials. The synergistic effect along with the flower-like morphology of the NRPC/NiMn electrode effectively increases the conductivity in two different ways: (i) through the formation of an increased number of electron conduction paths between NRPC/NiMn and CBZ and (ii) the interconnected flower-like morphology enables the attraction of the sensing material (CBZ) towards the interface of NRPC/NiMn [30,32]. The enhanced conductivity of NRPC/NiMn increases the detection level of CBZ, resulting in lower R_ct_ values, which is obvious from the increased current density signals obtained from CV curves (Figure 5b).

### 4.3. Effect of pH

As the prepared material, the NRPC/NiMn@GCE sensor is effective for the detection of CBZ; other factors, including pH, concentration of the sensing material, concentration of the analyte, scan rate, stability, interfering ion effect and real time sample analysis, had effects and the results are discussed in detail. The effect of pH for the detection of CBZ (30 µM) by the NRPC/NiMn (30 µM)@GCE sensor was analyzed using CV measurement and is depicted in Figure 5d. The relationship between the intensity of the peak current with respect to different pH is given in Figure 5e. PBS solution with 0.1 M concentration and different pH (3.0, 5.0, 7.0, 9.0 and 11.0) was taken for the CV measurement. As could be observed, the intensity of the peak current gradually increases up to pH 7 (0.8 mA g^−1^), and with further increases in pH (pH 9 and 11), there was a drastic decrease in the intensity of the current peak (0.19 mA g^−1^ at pH 9 and 0.17 mA g^−1^ at pH 11). The obtained results clearly show that the activity of the prepared sensor, NRPC/NiMn@GCE, can be fully utilized at neutral pH [30,32,33,34]. Either in acidic or basic environments, a decrease in activity was prominently found.

### 4.4. Effect of Material Concentration (NRPC/NiMn)

The effect of NRPC/NiMn concentration on CBZ detection was investigated by CV measurements. Five different concentrations of NRPC/NiMn (25, 50, 75, 100 and 125 µM) were used to fabricate the NRPC/NiMn@GCE sensors. As could be observed from Figure 5f, a clear separation of peaks is visible at the 50 µM concentration of NRPC/NiMn. The intensity of the anodic peak for CBZ was found to be 7.5 mA g^−1^ at the 50 µM concentration of NRPC/NiMn. The intensity of the peak increased gradually up to the 125 µM concentration of NRPC/NiMn with a higher current density of 15 mA g^−1^. A gradual increase in the intensity of the current signal even at a higher concentration of NRPC/NiMn (125 µM) is due to the fact that at a lower concentration of NRPC/NiMn (25 µM), the electrostatic attraction between NRPC/NiMn and CBZ occurs only at the surface of NRPC/NiMn due to the thin coating of the material. in contrast, st higher concentrations of NRPC/NiMn (125 µM), the electrostatic attraction occurs both at the surface and sub-surface of NRPC/NiMn, thereby producing increased current signals [31]. The linear regression equation (Figure 5g) for the anodic peak was found to be I_pa_ = 0.04543x + 3.4681 (R^2^ = 0.94378), and for the cathodic peak, it was found to be I_pc_ = −(0.01378x + 2.347) (R^2^ = 0.97625).

### 4.5. Effect of CBZ Concentration

The electrochemical sensing response of NRPC/NiMn@GCE for different concentrations of CBZ (0–50 µM) was determined using CV measurement in 0.1 M PBS solution at a pH of 7.0, and the results are depicted in Figure 5h. As can be observed from the figure, the intensity of the current peak shows a gradual increasing trend with increasing concentration of CBZ in the range of 0–50 µM. The current peak intensity for 40 and 50 µM of CBZ was very high, when compared with the other concentrations. A good linear relationship was obtained with the current peak value and CBZ concentrations (Figure 5i) and their corresponding regression equations were found to be I_pa_ = 0.4536x + 3.8767 (R^2^ = 0.9822) and I_pc_ = −(0.1377x + 2.4457) (R^2^ = 0.9511). The limit of detection was calculated using the following equation,

LOD = 3S/N(1)

where ‘LOD’ is the limit of detection; ‘S’ is the standard deviation of blank solution determination; and ‘N’ is the slope of the standard curve. The LOD value was found to be 25.64 µM in the linear detection range of CBZ 5 to 50 µM. Thus, the prepared NRPC/NiMn material is effective for the detection of CBZ [1,2,32].

### 4.6. Effect of Scan Rate

**Figure 6:**
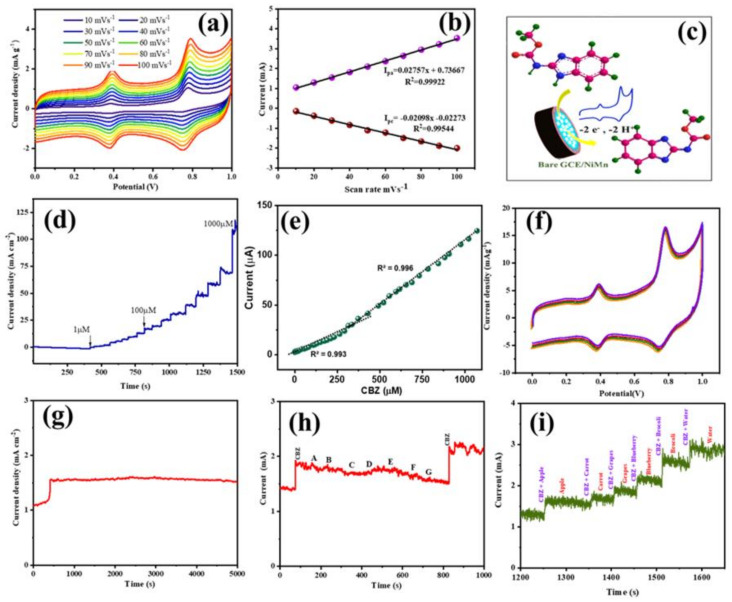
Electrochemical studies showing (**a**,**b**) effect of scan rate, (**c**) mechanism of carbendazim detection, (**d**,**e**) repeatability, (**f**) reproducibility, (**g**) stability and (**h**,**i**) anti-interfering ability of NRPC/NiMn.

CV measurement was used to analyze the effect of scan rate on the CBZ detection property using an NRPC/NiMn@GCE sensor. Figure 6a shows the CV curve at different scan rates from 10 to 100 mV s^−1^ in the potential range between 0 and 1.0 V. The figure displays that with increasing scan rate, both the anodic and cathodic peak current show a gradual increasing trend. Moreover, the plot of peak current versus scan rate (Figure 6b) shows a good linear relationship with a regression equation of I_pa_ = 0.0275x + 0.7366 (R^2^ = 0.9992), corresponding to the anodic peak and a regression equation of I_pc_ = 0.0209x + 0.0227 (R^2^ = 0.9954), corresponding to the cathodic peak, indicating an adsorption-controlled electrochemical process [30,32].

### 4.7. Repeatability, Reproducibility, Stability and Anti-Interfering Ability of NRPC/NiMn@GCE Sensor

The repeatability of the NRPC/NiMn@GCE sensor with different concentrations of CBZ was studied using amperometry, and the change in current obtained with respect to time was recorded and is depicted in Figure 6d. After each interval of time, the concentration of CBZ varied between 1 and 1000 µM. It can be observed from the figure that during each CBZ addition, there was a rise in the current peak, which was maintained for a longer period of time. Obviously, the current peak increased only with the addition of CBZ at each step. Figure 6e shows the plot of current obtained versus CBZ concentration. As can be observed, a linear range is obtained with a slight deviation at lower CBZ concentrations. A relatively lower % of RSD value, less than 3%, was obtained, showing acceptable repeatability of the NRPC/NiMn@GCE sensor for CBZ detection.

The reproducibility of NRPC/NiMn@GCE sensor for 30 µM CBZ detection was studied using CV measurements. Five different NRPC/NiMn@GCE sensors were prepared uniformly, without changing either their concentration or preparation method. CV measurement was performed for these five electrode materials, maintaining the CBZ concentration of 30 µM, and the obtained CV curve is presented in Figure 6f. The CV curves do not show any appreciable change for these five sensor materials, which proves the reproducibility of the NRPC/NiMn@GCE sensor.

The stability of the NRPC/NiMn@GCE sensor for CBZ detection was analyzed by amperometry and is depicted in Figure 6g. The amperometry result shows that the response of NRPC/NiMn@GCE sensor for 30 µM of CBZ gives a current peak at 1.5 mA cm^−2^, which was maintained for a longer period of time (up to 5000 s). Moreover, it was found that the current response signal was retained up to 96.6%, when compared with its initial value.

To evaluate the anti-interference ability of the prepared NRPC/NiMn@GCE sensor, 0.1 M PBS solution containing 30 µM of CBZ was used. Some common interfering ions (Na^+^, Ag^+^, K^+^, Zn^2+^, Ca^2+^, Mg^2+^ and Ni^2+^) with 1000 µM concentration were added in specific time intervals, and any changes in the current value were monitored and are displayed in Figure 6h. The results indicated that even with increased concentration of the common ions (concentration of common interfering ions is almost 33 times higher than CBZ concentration), no obvious increase in the current peak could be observed. This indicates excellent selectivity of the NRPC/NiMn@GCE sensor for CBZ detection, without the interference of the added co-existing ions [1,2,30,32]. A similar observation was obtained for the anti-interfering ability of the real samples (Figure 6i). The selectivity of the sensor for CBZ in the presence of other pesticides will be evaluated in our future work.

### 4.8. CBZ Detection in Real Samples

**Figure 7:**
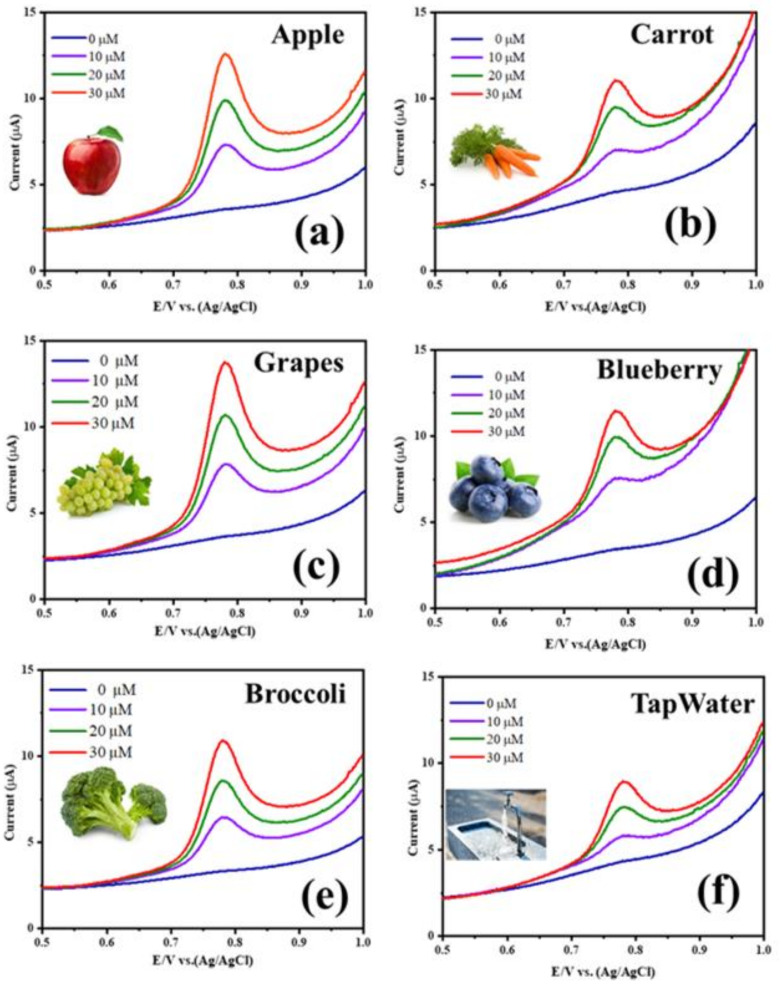
Real-time analysis of NRPC/NiMn sensor: (**a**) Apple, (**b**) Carrot, (**c**) Grapes, (**d**) Blueberry, (**e**) Broccoli and (**f**) Tap water.

The practical feasibility of the NRPC/NiMn@GCE sensor was validated by quantifying the CBZ detection in real samples, such as apple, carrot, grapes, blueberry and broccoli, and in environmental sample, such as tap water. The real samples were obtained from the supermarket and the tap water was taken from the laboratory. The extract of each real samples was obtained by chopping them into small pieces, grinding without adding any water, and finally filtering to obtain their extracts. In the case of apple, carrot, grapes and blueberries, the samples were taken along with the skin, crushed well and filtered to obtain their extract. In the case of broccoli, the flower part attached with the stalk was taken, crushed well and the extract was taken after filtration. In the case of tap water, it was taken as such. About 10 mL of each sample was taken separately, to which different concentrations of CBZ (0, 10, 20 and 30 µM) were added and DPV measurement was taken to analyze the concentration of the pesticide. Figure 7 displays the DPV curves of the samples without any spiked CBZ and a known quantity of spiked CBZ (10, 20 and 30 µM). The DPV curves of all the samples (pure extract without spiking CBZ) do not show any observable peak for CBZ detection, which demonstrates that in the real sample, the concentration of pesticide is very low, and is undetectable without adding a known concentration of CBZ. All the samples spiked with CBZ display a clear peak around 0.8 V, indicating the presence of CBZ. Moreover, with increased concentration of CBZ (from 10 µM to 30 µM), the intensity of the current peak is also increased. From the concentration of CBZ obtained from spiked CBZ and found CBZ, their percentage recovery was determined and the values are tabulated in Table 1. The % recoveries of CBZ were found to be in the range of 97.29–102.69% for apple, 99.83–100.16% for carrot, 96.94–103.05% for broccoli, 96.40–103.6% for grapes, 95.87–104.98% for blueberries and 98.81–101.19% for tap water, exhibiting good accuracy of the NRPC/NiMn@GCE sensor for CBZ detection. Moreover, for all the samples, their obtained RSD values were very low, between 0.66 and 2.01% for apple, 0.05 and 0.14% for carrot, 0.71 and 2.15% for broccoli, 0.84 and 2.59% for grapes, 1.21 and 3.45% for blueberries and 0.25 and 0.85% for tap water, indicating the sensitive CBZ detection of the NRPC/NiMn@GCE sensor [32,35,36]. The obtained results confirm a promising practical applicability of the prepared sensor for CBZ detection (Table 2). Thus, a simple strategy would be adopted using polybenzoxazine carbon and bimetal oxides, eliminating the use of expensive materials, such as gold, gold nanoparticles, palladium and multiwalled carbon nanotubes.

## 5. Conclusions

In summary, a novel NRPC/NiMn@GCE sensor was successfully fabricated and explored for the first time for the quantitative electrochemical detection of carbendazim. The synergistic effect between nitrogen-rich porous carbon, and bimetals comprised of nickel and manganese improved the electrochemical performance by enhancing the current response for CBZ, when compared with other materials. Interestingly, benefiting from the porous nature, flower-like morphology and highly conductive interconnected NRPC/NiMn network, strong adherence of CBZ with NRPC/NiMn was achieved. Under optimal conditions, the NRPC/NiMn@GCE sensor provides a favorable linear range of detection from 5 to 50 mM, exhibiting excellent sensitivity, stability, repeatability and anti-interfering ability. Furthermore, the real-time monitoring of the NRPC/NiMn@GCE sensor was proved by the successful determination of CBZ in fruits, vegetables and tap water with satisfactory recovery between 96.40 and 104.98%. Thus, our research work demonstrates a feasible strategy for the development of a highly efficient electrochemical sensor for the detection of pesticides that can be applied practically.

## Figures and Tables

**Table 1:** Data obtained from real-time analysis of NRPC/NiMn sensor.

Real Samples	CBZ Spiked (µM)	CBZ Found (µM)	Recovery (%)	RSD (%)
**Apple**	10	4.86	97.29	2.01
	20	10.27	102.69	1.88
	30	14.86	99.09	0.66
**Carrot**	10	4.99	99.83	0.14
	20	10.02	100.16	0.14
	30	14.99	99.94	0.05
**Broccoli**	10	4.85	96.94	2.15
	20	10.30	103.05	2.09
	30	14.85	98.98	0.71
**Grapes**	10	5.18	103.6	2.5
	20	9.64	96.40	2.59
	30	15.18	101.2	0.84
**Blueberry**	10	5.25	104.98	3.45
	20	9.83	101.29	1.21
	30	14.38	95.87	2.98
**Tap water**	10	5.06	101.19	0.84
	20	9.88	98.81	0.85
	30	15.05	100.39	0.25

**Table 2:** A comparison table showing the electrochemical sensing ability of various sensors for CBZ.

Sensor Type	Electrode Material	Detection Method	Limit of Detection (LOD)	Linear Range	Reference
Au-ZrO2-GNs/GCE	Gold and zirconia nanocomposites on graphene sheets	Electrochemical detection	0.001 µM	-	[5]
f-MWCNT/AgNPs	Functionalized multi-walled carbon nanotubes/silver	Electrochemical detection	0.2778 mM	0–8 mM	[6]
Pd-MWCNTs	Palladium-modified multi-walled carbon nanotubes	Electrochemical detection	0.13 µM	0.5–100 µM	[17]
NPG/GC	Nano-porous gold on glassy carbon	Electrochemical detection	0.24 mM	-	[20]
NRPC/NiMn@GCE	Nitrogen-rich porous carbon (NRPC) with Ni & Mn	Electrochemical detection	25.64 µM	5–50 µM	[this work]

## Data Availability

The original contributions presented in the study are included in the article/Supplementary Materials, further inquiries can be directed to the corresponding author.

## Supplementary Materials

The following supporting information can be downloaded at: https://www.mdpi.com/article/10.3390/bios14100474/s1. Scheme S1: Synthesis of benzoxazines monomer (HPh-Bzo) and NRPC. Figure S1: (a) Raman, (b) XRD, (c) N2 adsorption desorption and (d) pore size distribution of the synthesized materials [a-NRPC, b-NRPC/Mn, c-NRPC/Ni and d-NRPC/NiMn].

## References

[1] Yukird J., Insin N., Chanajaree R., Rodthongkum N. (2023). Fe_3_O_4_ Nanoparticle/Graphene Oxide Composites as Selective Probes and Self-Matrixes for Pesticide Detection by Electrochemistry and Laser Desorption/Ionization Mass Spectrometry. ACS Appl. Nano Mater..

[2] Liu Y., Wu T., Zhao H., Zhu G., Li F., Guo M., Ran Q., Komarneni S. (2023). An Electrochemical Sensor Modified with Novel Nanohybrid of Super-P Carbon Black@zeolitic-Imidazolate-Framework-8 for Sensitive Detection of Carbendazim. Ceram. Int..

[3] Lambraki I.A., Chadag M.V., Cousins M., Graells T., Leger A., Henriksson P.J.G., Troell M.F., Harbarth S., Wernli D., Jorgensen P.S. (2023). Factors Impacting Antimicrobial Resistance in the South East Asian Food System and Potential Places to Intervene: A Participatory, One Health Study. Front. Microbiol..

[4] Chen M., Zhao Z., Lan X., Chen Y., Zhang L., Ji R., Wang L. (2015). Determination of Carbendazim and Metiram Pesticides Residues in Reapeseed and Peanut Oils by Fluorescence Spectrophotometry. Meas. J. Int. Meas. Confed..

[5] Eissa S., Zourob M. (2017). Selection and Characterization of DNA Aptamers for Electrochemical Biosensing of Carbendazim. Anal. Chem..

[6] Cabrera L.C., Pastor P.M., EFSA (European Food Safety Authority) (2022). The 2020 European Union report on pesticide residues in food. EFSA J..

[7] Wang K., Sun D.W., Pu H., Wei Q. (2020). A Rapid Dual-Channel Readout Approach for Sensing Carbendazim with 4-Aminobenzenethiol-Functionalized Core-Shell Au@Ag Nanoparticles. Analyst.

[8] Singh S., Singh N., Kumar V., Datta S., Wani A.B., Singh D., Singh K., Singh J. (2016). Toxicity, Monitoring and Biodegradation of the Fungicide Carbendazim. Environ. Chem. Lett..

[9] Huan Z., Luo J., Xu Z., Xie D. (2016). Acute Toxicity and Genotoxicity of Carbendazim, Main Impurities and Metabolite to Earthworms (*Eisenia foetida*). Bull. Environ. Contam. Toxicol..

[10] Özcan A., Hamid F., Özcan A.A. (2021). Synthesizing of a Nanocomposite Based on the Formation of Silver Nanoparticles on Fumed Silica to Develop an Electrochemical Sensor for Carbendazim Detection. Talanta.

[11] Zhao H., Ran Q., Li Y., Li B., Liu B., Ma H., Zhang M., Komarneni S. (2020). Highly Sensitive Detection of Gallic Acid Based on 3D Interconnected Porous Carbon Nanotubes/Carbon Nanosheets Modified Glassy Carbon Electrode. J. Mater. Res. Technol..

[12] Guo Y., Guo S., Li J., Wang E., Dong S. (2011). Cyclodextrin-Graphene Hybrid Nanosheets as Enhanced Sensing Platform for Ultrasensitive Determination of Carbendazim. Talanta.

[13] Wang L., Li G., Wu X., Xu P. (2016). Comparative Proteomic Analyses Provide Novel Insights into the Effects of Grafting Wound and Hetero-Grafting per Se on Bottle Gourd. Sci. Hortic..

[14] Radisic M., Grujic S., Vasiljevic T., Lausevic M. (2009). Determination of Selected Pesticides in Fruit Juices by Matrix Solid-Phase Dispersion and Liquid Chromatography-Tandem Mass Spectrometry. Food Chem..

[15] Pourreza N., Rastegarzadeh S., Larki A. (2015). Determination of Fungicide Carbendazim in Water and Soil Samples Using Dispersive Liquid-Liquid Microextraction and Microvolume UV-Vis Spectrophotometry. Talanta.

[16] Zhu C., Liu D., Chen Z., Li L., You T. (2019). An Ultra-Sensitive Aptasensor Based on Carbon Nanohorns/Gold Nanoparticles Composites for Impedimetric Detection of Carbendazim at Picogram Levels. J. Colloid Interface Sci..

[17] Gao N., He C., Ma M., Cai Z., Zhou Y., Chang G., Wang X., He Y. (2019). Electrochemical Co-Deposition Synthesis of Au-ZrO2-Graphene Nanocomposite for a Nonenzymatic Methyl Parathion Sensor. Anal. Chim. Acta.

[18] Anshori I., Nuraviana Rizalputri L., Rona Althof R., Sean Surjadi S., Harimurti S., Gumilar G., Yuliarto B., Handayani M. (2021). Functionalized Multi-Walled Carbon Nanotube/Silver Nanoparticle (f-MWCNT/AgNP) Nanocomposites as Non-Enzymatic Electrochemical Biosensors for Dopamine Detection. Nanocomposites.

[19] Fu S., Ma X., Wang S., Zha Q., Wen W., Hu B. (2021). Surfactant-Assisted Carbon Black for the Electrochemical Detection of Endocrine Disruptors. Surf. Interfaces.

[20] Wang Z., Liu Y., Li F., Dubovyk V., Guo M., Zhu G., Ran Q., Zhao H. (2022). Electrochemical Sensing Platform Based on Graphitized and Carboxylated Multi-Walled Carbon Nanotubes Decorated with Cerium Oxide Nanoparticles for Sensitive Detection of Methyl Parathion. J. Mater. Res. Technol..

[21] Ji J., Zhou Z., Zhao X., Sun J., Sun X. (2015). Electrochemical Sensor Based on Molecularly Imprinted Film at Au Nanoparticles-Carbon Nanotubes Modified Electrode for Determination of Cholesterol. Biosens. Bioelectron..

[22] Wu Y., Wu Y., Lv X., Lei W., Ding Y., Chen C., Lv J., Feng S., Chen S.M., Hao Q. (2020). A Sensitive Sensing Platform for Acetaminophen Based on Palladium and Multi-Walled Carbon Nanotube Composites and Electrochemical Detection Mechanism. Mater. Chem. Phys..

[23] Gao X., Gao Y., Bian C., Ma H., Liu H. (2019). Electroactive Nanoporous Gold Driven Electrochemical Sensor for the Simultaneous Detection of Carbendazim and Methyl Parathion. Electrochim. Acta.

[24] Li D., Zhao H., Wang G., Rinklebe J., Lam S.S., Liu R., Bai L. (2022). Ultrasensitive Determination of Diquat Using a Novel Nanohybrid Sensor Based on Super-P Nanoparticles Dispersed Palygorskite Nanofibers. Sens. Actuators B Chem..

[25] Li K., Kang J., Zhan T., Cao W., Liu X., Gao H., Si C., She X. (2021). Electrochemical Sensing Platform for Naphthol Isomers Based on in Situ Growth of ZIF-8 on Reduced Graphene Oxide by a Reaction-Diffusion Technique. J. Colloid Interface Sci..

[26] Zhang W., Zong L., Liu S., Pei S., Zhang Y., Ding X., Jiang B., Zhang Y. (2019). An Electrochemical Sensor Based on Electro-Polymerization of Caffeic Acid and Zn/Ni-ZIF-8–800 on Glassy Carbon Electrode for the Sensitive Detection of Acetaminophen. Biosens. Bioelectron..

[27] Nie M., Lu S., Lei D., Yang C., Zhao Z. (2017). Rapid Synthesis of ZIF-8 Nanocrystals for Electrochemical Detection of Dopamine. J. Electrochem. Soc..

[28] Li R., Ren X., Feng X., Li X., Hu C., Wang B. (2014). A Highly Stable Metal- and Nitrogen-Doped Nanocomposite Derived from Zn/Ni-ZIF-8 Capable of CO2 Capture and Separation. Chem. Commun..

[29] Kim J., Kang I., Kim S., Kang J. (2018). Facile synthesis of partially oxidized Mn3O4-functionalized carbon cathodes for rechargeable Li–O2 batteries. RSC Adv..

[30] Yao L., Zhou C., Hu N., Hu J., Hong M., Zhang L., Zhang Y. (2018). Flexible Graphene/Carbon Nanotube Hybrid Papers Chemical-Reduction-Tailored by Gallic Acid for High-Performance Electrochemical Capacitive Energy Storages. Appl. Surf. Sci..

[31] Dong Y., Yang L., Zhang L. (2017). Simultaneous Electrochemical Detection of Benzimidazole Fungicides Carbendazim and Thiabendazole Using a Novel Nanohybrid Material-Modified Electrode. J. Agric. Food Chem..

[32] Carpenter C.R., Shipway P.H., Zhu Y., Weston D.P. (2011). Effective Dispersal of CNTs in the Fabrication of Electrodeposited Nanocomposites. Surf. Coat. Technol..

[33] Kokulnathan T., Chen S.M. (2020). Design and Construction of the Gadolinium Oxide Nanorod-Embedded Graphene Aerogel: A Potential Application for Electrochemical Detection of Postharvest Fungicide. ACS Appl. Mater. Interfaces.

[34] Zhu G., Yi Y., Sun H., Wang K., Han Z., Wu X. (2015). Cyclodextrins functionalized hollow carbon nanospheres by introducing nanogold for enhanced electrochemical sensing of o-dihydroxybenzene and p-dihydroxybenzene. J. Mater. Chem. B.

[35] Han H.S., You J.M., Seol H., Jeong H., Jeon S. (2014). Electrochemical Sensor for Hydroquinone and Catechol Based on Electrochemically Reduced GO-Terthiophene-CNT. Sens. Actuators B Chem..

[36] Zhang Q., Zhang Z., Xu S., Liu A., Da L., Lin D., Jiang C. (2023). Photoinduced Electron Transfer-Triggered g-C3N4/Rhodamine B Sensing System for the Ratiometric Fluorescence Quantitation of Carbendazim. Anal. Chem..

