# Supplementary material for: Advanced Electrochemical Monitoring of Carbendazim Fungicide in Foods Using Interfacial Superassembly of NRPC/NiMn Frameworks
Source: Biosensors (Basel). 2024 Oct 2;14(10):474. doi: 10.3390/bios14100474 (PMC11505953; doi:10.3390/bios14100474)
Supplement: Supplementary file 1 [file biosensors-14-00474-s001.zip › biosensors-3208063-supplementary.pdf]

biosensors

MDPI

Supporting Information

Advanced Electrochemical Monitoring of Carbendazim Fungicide in Foods Using Interfacial Superassembly of NRPC/NiMn Frameworks

Materials

Phenylethylamine, hydroquinone, and paraformaldehyde were procured from Sigma-Aldrich (USA), while melamine sponge, stearic acid, potassium hydroxide (KOH), sodium hydroxide (NaOH), dimethyl sulfoxide (DMSO), nickel nitrate hexahydrate, manganese nitrate tetrahydrate, polyvinylidene fluoride (PVDF), and N, N-dimethylformamide (DMF) were obtained from Duksan Chemicals Co., Ltd. in the Republic of Korea. These chemicals were used as received, without any further purification.

Synthesis of benzoxazines monomer (HPh-Bzo)

In a three-necked round-bottom flask fitted with a magnetic stirrer and a reflux condenser, 18.0 grams (0.6 mol) of paraformaldehyde was placed along with 100 mL of dimethyl sulfoxide (DMSO). The mixture was stirred at 70°C. Concurrently, a separate solution was prepared by dissolving 24.2 grams (0.2 mol) of phenylethylamine in DMSO while stirring. This phenylethylamine solution was added dropwise to the paraformaldehyde–DMSO mixture. Meanwhile, a solution containing 11.0 grams (0.1 mol) of hydroquinone in 20 mL of DMSO was prepared. Upon the complete addition of phenylethylamine, the hydroquinone solution was added dropwise to the reaction mixture. The temperature was then gradually increased to 120 °C and maintained at this level for 5 hours while stirring continuously. At the end of the reaction time, a transparent pale yellow solution was obtained. The solution was cooled to room temperature and precipitated in a 1N NaOH solution. The resulting precipitate was washed several times with distilled water, filtered, and finally dried under vacuum at 60 °C for 12 hours to obtain the HPh-Bzo monomer (Scheme S1).

Synthesis of NRPCs

The preparation of nitrogen-rich porous carbons (NRPCs) involved several steps to ensure the formation of a well-defined porous structure free of impurities. Initially, the HPh-Bzo monomer underwent curing/self-polymerization followed by carbonization and activation. Curing was achieved through a stepwise heating process in an oven, with temperatures ranging from 100 to 250 °C, each maintained for 4 hours. This process resulted in the formation of polybenzoxazine, the polymer precursor. Subsequently, carbonization was carried out by heating the polybenzoxazine at 600 °C under an Ar atmosphere for 5 hours with a heating rate of 1 °C/min. This step led to the conversion of the polymer into carbon material. To further enhance the porosity, the carbonized material was soaked in an aqueous KOH solution overnight with a weight ratio of KOH to carbonized sample of 2:1. After filtration, the sample was dried at 120 °C. Activation of the dried sample was then performed by heating it at 800 °C in an Ar atmosphere with a ramp rate of 3 °C/min using a tube furnace. This step contributed to the development of the porous structure.

Following activation, the products underwent washing with 1M HCl and deionized water until a neutral pH was achieved. Subsequently, the sample was dried at 110 °C for 12 hours. The resulting dried sample was designated as an NRPC sample (Scheme S1) with a yield of 42%. This process ensures the production of nitrogen-rich porous carbons with high purity and well-defined porosity.

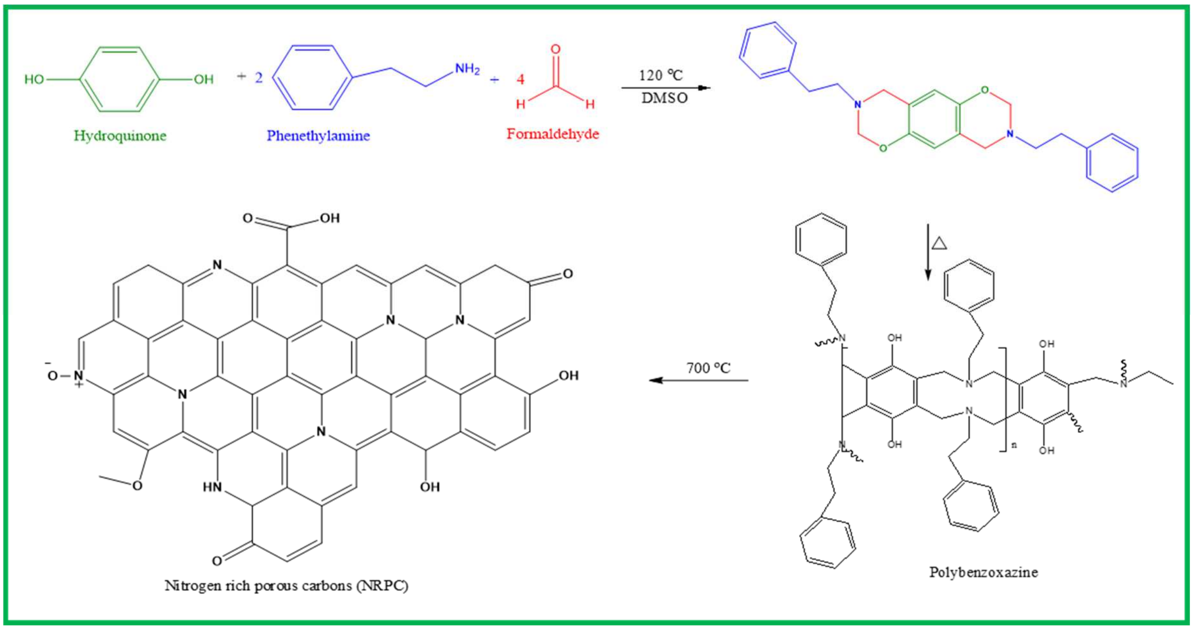

Scheme S1. Synthesis of benzoxazines monomer (HPh-Bzo) and NRPC.

Instrumentation methods

Prepared composite materials underwent comprehensive characterization using a variety of physicochemical techniques. Field emission scanning electron microscopy (FESEM) combined with energy-dispersive X-ray spectroscopy (EDS) provided detailed surface morphology and elemental composition analysis. FESEM with EDS analysis was conducted using a Hitachi S-4800 instrument operating at an accelerating voltage of 4 kV. High-resolution transmittance electron microscopy (HRTEM) was employed to investigate the internal structure and morphology of the materials. HRTEM images were acquired using an FEI-Tecnai TF-20 transmission electron microscope with an operating accelerating voltage of 120 kV. X-ray diffraction (XRD) measurements were performed to analyze the crystalline structure of the materials. XRD patterns were obtained using a PANalytical X′Pert3 MRD diffractometer with monochromatized Cu Kα radiation (λ = 1.54 Å) at 40 kV and 30 mA over the 2θ range from 10 to 80°. Raman spectroscopy was utilized to assess the molecular structure and chemical composition of the composites. Raman spectra were recorded using an XploRA Micro-Raman spectrophotometer (Horiba) within the range of 500 to 4000 cm^{-1}. Nitrogen adsorption–desorption isotherms were measured to determine the surface area and pore characteristics of the materials. The measurements were carried out at -197 °C using a Micromeritics ASAP 2000 instrument after the samples were dried at 120 °C and evacuated for 8 hours in flowing argon at 140 °C. Surface area, pore size, and pore volumes were calculated from the isotherms using the Brunauer–Emmet–Teller (BET) and Barrett–Joyner–Halenda (BJH) equations. Attenuated total reflectance Fourier-transform infrared (ATR-FTIR) spectroscopy was employed to investigate the functional groups present in the materials. X-ray photoelectron spectroscopy (XPS) was utilized to analyze the surface chemistry and elemental composition of the composites. XPS spectra were obtained using a K-Alpha instrument (Thermo Scientific), and CasaXPS software was employed for the deconvolution of the high-resolution XPS spectra. All of these analyses were conducted at the core research support center for natural products and medical materials of Yeungnam University.

Fabrication of working electrodes and their electrochemical measurements

Various electrochemical experiments were conducted employing cyclic voltammetry, amperometric (i-t) analysis, and impedance spectroscopy. These experiments were carried out using a corrtest potentiostat/galvanostat electrochemical workstation manufactured

in China. The traditional three-electrode system utilized consisted of a glassy carbon electrode (GCE) as the working electrode, a saturated KCl-based Ag/AgCl electrode as the reference, and a platinum wire as the counter electrode. These electrodes were immersed in a phosphate-buffer solution (pH 7.0).

The impedance studies were performed using electrochemical impedance spectroscopy (EIS). All measurements were conducted at room temperature.

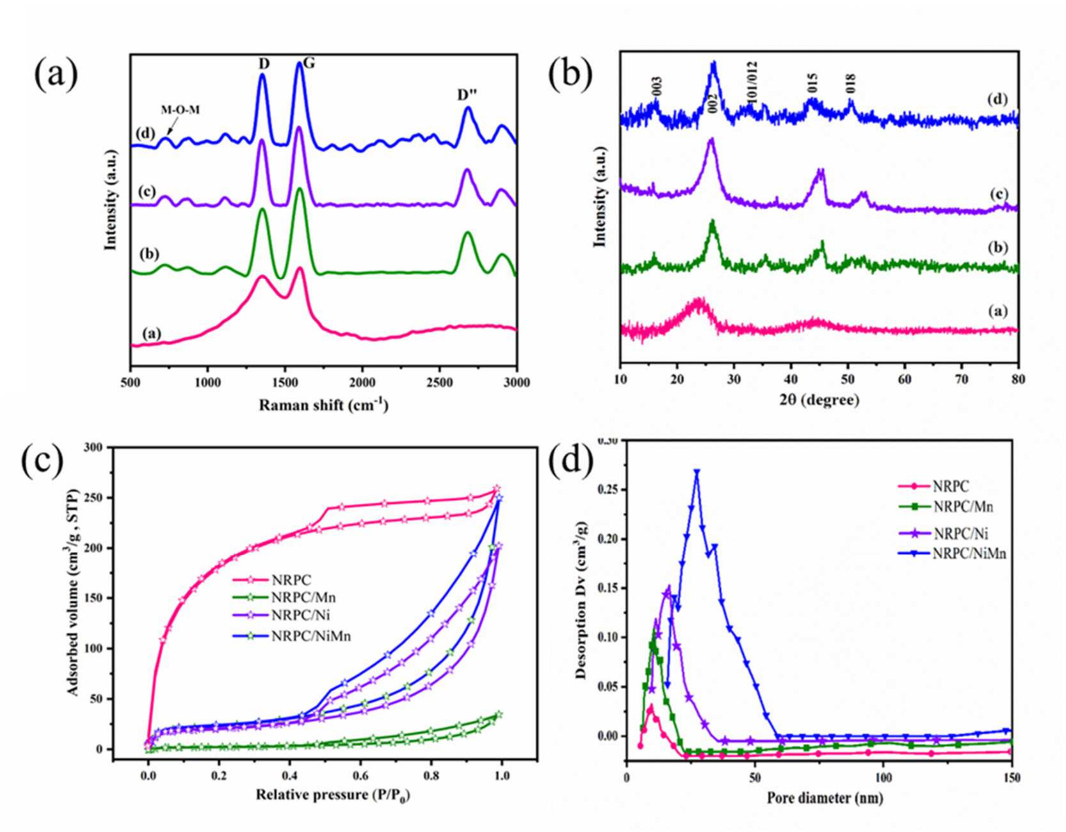

Figure S1. (a) Raman, (b) XRD, (c) N_2 adsorption desorption, and (d) pore size distribution of the synthesized materials [a-NRPC, b-NRPC/Mn, c-NRPC/Ni, and d-NRPC/NiMn].
